# Comparing Effect Estimates in Randomized Trials and Observational Studies From the Same Population: An Application to Percutaneous Coronary Intervention

**DOI:** 10.1161/JAHA.120.020357

**Published:** 2021-05-15

**Authors:** Anthony A. Matthews, Karolina Szummer, Issa J. Dahabreh, Bertil Lindahl, David Erlinge, Maria Feychting, Tomas Jernberg, Anita Berglund, Miguel A. Hernán

**Affiliations:** ^1^ Unit of Epidemiology Institute of Environmental Medicine Karolinska Institutet Stockholm Sweden; ^2^ Department of Cardiology Karolinska University Hospital Stockholm Sweden; ^3^ Department of Medicine Karolinska Institutet Huddinge Sweden; ^4^ Department of Biostatistics Harvard T.H. Chan School of Public Health Boston MA; ^5^ Department of Epidemiology Harvard T.H. Chan School of Public Health Boston MA; ^6^ Department of Medical Sciences, Cardiology and Uppsala Clinical Research Center Uppsala University Uppsala Sweden; ^7^ Department of Cardiology Clinical Sciences Lund University Skåne University Hospital Lund Sweden; ^8^ Department of Clinical Sciences Danderyd University Hospital‐Karolinska Institute Danderyd Sweden; ^9^ Harvard‐MIT Division of Health Sciences and Technology Boston MA

**Keywords:** methodology, observational studies, randomized controlled trials, registry, SWEDEHEART, target trial emulation, Anticoagulants, Percutaneous Coronary Intervention, Cardiovascular Disease

## Abstract

**Background:**

To understand when results from observational studies and randomized trials are comparable, we performed an observational emulation of a target trial designed to ask similar questions as the VALIDATE (Bivalirudin Versus Heparin in ST‐Segment and Non–ST‐Segment Elevation Myocardial Infarction in Patients on Modern Antiplatelet Therapy) randomized trial. The VALIDATE trial compared the effect of bivalirudin and heparin during percutaneous coronary intervention on the risk of death, myocardial infarction, and bleeding across Sweden.

**Methods and Results:**

We specified the protocol of a target trial similar to the VALIDATE trial, then emulated the target trial in the period before the VALIDATE trial took place using data from the SWEDEHEART (Swedish Web System for Enhancement and Development of Evidence‐Based Care in Heart Disease Evaluated According to Recommended Therapies) registry—the same registry in which the trial was undertaken. The target trial emulation and the VALIDATE trial both estimated little or no effect of bivalirudin versus heparin on the risk of death or myocardial infarction by 180 days (target trial emulation risk ratio for death, 1.21 [95% CI, 0.88 – 1.54]; VALIDATE trial hazard ratio for death, 1.05 [95% CI, 0.78 – 1.41]). The observational data, however, could not capture less severe cases of bleeding, resulting in an inability to define a bleeding outcome like the trial, and could not accurately estimate the comparative risk of death by 14 days, which may be the result of intractable confounding early in follow‐up or the inability to precisely emulate the trial’s eligibility criteria.

**Conclusions:**

Using real‐world data to emulate a target trial can deliver accurate effect estimates. Yet, even with rich observational data, it is not always possible to estimate the short‐term effect of interventions or the effect on outcomes for which data are not routinely collected.

**Nonstandard Abbreviations and Acronyms**
RIKS‐HIA: Register of Information and Knowledge About Swedish Heart Intensive Care Admissions
SCAAR: Swedish Coronary Angiography and Angioplasty Registry
SEPHIA: Swedish Heart Surgery Register and the National Registry of Secondary Prevention
SWEDEHEART: Swedish Web System for Enhancement and Development of Evidence‐Based Care in Heart Disease Evaluated According to Recommended Therapies
VALIDATE: Bivalirudin Versus Heparin in ST‐Segment and Non–ST‐Segment Elevation Myocardial Infarction in Patients on Modern Antiplatelet Therapy

**Clinical Perspective**

**What is New?**

- Using observational data to emulate a target trial can deliver accurate estimates for the comparative effect of bivalirudin and heparin during percutaneous coronary intervention on the risk of death and myocardial infarction by 180 days.
- Observational data cannot be used to answer all clinical questions as study‐specific information is not always routinely collected. In this instance, it was not possible to accurately estimate the effect on the risk of bleeding or the short‐term effect on the risk of death.

**What are the Clinical Implications?**

- Synergy between randomized trials and observational analyses of real‐world data has the potential to revolutionize the length of time patients can be followed up and the speed at which results from trials reach clinical practice.

Observational analyses of routinely collected healthcare data, a form of “real‐world evidence,” are often used to evaluate the benefits and risks of clinical interventions for acute coronary syndromes. Most criticisms of these observational analyses revolve around the lack of randomized assignment of the treatment strategies under comparison, which may result in confounded effect estimates.^1^, ^2^, ^3^ Despite this limitation, routinely collected healthcare data can be used to explicitly emulate target trials, as recent applications in diverse clinical areas have shown.^4^, ^5^, ^6^, ^7^, ^8^

Routinely collected observational data can only be used to emulate pragmatic trials, not trials with placebo control and blind treatment assignment, or those that rely on detailed data collection to determine eligibility or ascertain outcomes.^9^ Specifically, the lack of detailed information on eligibility criteria and outcomes restricts the type of causal questions that can be answered using real‐world data, a point that is not always emphasized in discussions about the topic.^4^

To study the strengths and limitations of observational analyses that emulate a trial, a near‐ideal scenario is to compare a registry‐based trial versus the emulation of a target trial with the same protocol as the registry‐based trial using observational data from the same registry.^10^ This comparison ensures that the causal question is asked in the same population and healthcare setting.^11^ SWEDEHEART (Swedish Web System for Enhancement and Development of Evidence‐Based Care in Heart Disease Evaluated According to Recommended Therapies) is a national quality registry of myocardial infarction (MI), coronary intervention, and heart surgery, which includes longitudinal information on demographic and clinical characteristics, use of therapeutic and preventive services, diagnostic procedures, and various measures of healthcare utilization for the whole of Sweden.^12^ By using the registry to enroll participants, randomize interventions, and report outcomes, SWEDEHEART can be used to run trials nested within the registry.^13^ An example of a registry‐based trial within SWEDEHEART is the VALIDATE (Bivalirudin Versus Heparin in ST‐Segment and Non–ST‐Segment Elevation Myocardial Infarction in Patients on Modern Antiplatelet Therapy) trial, which compared 2 anticoagulation interventions, bivalirudin and heparin, over 180 days in patients with acute MI undergoing percutaneous coronary intervention (PCI) in Sweden.^14^

Here, we use the observational data from the SWEDEHEART registry to emulate a target trial designed to answer similar questions as the VALIDATE trial, and we outline conditions under which one can successfully design a target trial and emulate it using observational data. We describe the VALIDATE trial and the observational data from the SWEDEHEART registry, the specification of the target trial, and the emulation procedures. Our example illustrates the opportunities and limitations of healthcare registries to emulate a target trial.

## VALIDATE: The Index Trial

### Trial Design and Analysis

The VALIDATE trial was a multicenter, randomized, controlled, open‐label clinical trial performed between June 2014 and September 2016. Individuals in the SWEDEHEART registry were eligible to participate if they were admitted to the hospital with a diagnosis of ST‐segment–elevation MI (STEMI) or non–ST‐segment–elevation MI (NSTEMI), urgent PCI was planned in 1 of 25 of the 29 PCI centers in Sweden, and some other eligibility criteria were met (Table 1). Individuals who accepted the invitation to participate were randomly assigned to receive either intravenous bivalirudin (0.75 mg/kg) or intra‐arterial unfractionated heparin (5000 U/mL) under PCI intervention. The primary end point was the composite of death from any cause, MI, or major bleeding by 180 days. The individual components of the composite event were also assessed. Research nurses screened for clinical end point events by contacting the patients or first‐degree relatives by telephone 7 days and 180 days after PCI. If the patient or relatives could not be contacted after repeated telephone calls and a mailed letter, information was collected through review of hospital records. The intention‐to‐treat analysis, described in detail elsewhere, relied on the comparison of 180‐day risk differences and hazard ratios (HRs).^14^

**Table 1:** Description of the VALIDATE Randomized Trial, Target Trial, and Target Trial Emulation Using SWEDEHEART Register

Protocol Component	VALIDATE Trial	Target Trial	Target Trial Emulation Using SWEDEHEART
Eligibility criteria	Age ≥18 years between June 2014 and September 2016 Diagnosis of NSTEMI as defined by guidelines (positive troponin) or STEMI as defined by chest pain for at least 30 minutes and an ECG with new ST‐segment elevation in ≥2 contiguous leads of ≥0.2 mV in leads V2–V3 and/or ≥0.1 mV in other leads or a probable new‐onset left bundle branch block Therapeutic PCI (not primarily diagnostic PCI) after angiography in 1 of 25 (of 29) selected clinics Ability to provide informed consent: In patients with STEMI, witnessed oral consent was obtained after angiography and before randomization. Within the following 24 h, after written information about the trial had been provided, the patients confirmed further participation by providing written informed consent. In patients with NSTEMI, written consent was obtained before angiography. Treated with a bolus dose of ticagrelor, prasugrel, or cangrelor before start of PCI Treated with aspirin in accordance with local practices Life expectancy >1 y Patients with known ongoing bleeding No heparin >5000 U before arriving to PCI laboratory. Up to 3000 U of heparin allowed during angiography. No uncontrolled hypertension, subacute bacterial endocarditis, severe renal (GFR <30 mL/min) and/or liver dysfunction, thrombocytopenia or thrombocyte function defects No contraindication for the study medications No glycoprotein IIb/IIIa inhibitors given or are preplanned to be given during the procedure	Same as VALIDATE apart from: Study period between January 2012 and May 2014 Without asking for informed consent Known terminal disease defined as palliative care, dialysis, dementia, or aggressive cancer (pancreatic, mesothelioma, lung, liver, stomach, or brain) in 3 y before PCI Unable to identify ongoing bleeding No information on dose of heparin before PCI, so excluded all individuals given any prior heparin Unable to identify contraindications to study medications Individuals who died on the day of PCI were excluded and identification of outcomes start from day after PCI	Same as target trial
Treatment strategies	(1) Bivalirudin: intravenous bolus of 0.75 mg/kg of body weight followed by an infusion of 1.75 mg/kg per hour. Treatment was started as soon as PCI of the culprit lesion was planned. Continuation of the bivalirudin infusion after PCI until completion of the last vial is strongly recommended. (2) Heparin: 70 to 100 U/kg	(1) Individual administered bivalirudin under PCI (2) Individual administered heparin under PCI If the patient was given both bivalirudin and heparin, then it was assumed that heparin was low‐dose intra‐arterial administration of up to 3000 U, and the treatment strategy was therefore bivalirudin, as no information was available on dosage.	Same as target trial. Individuals were classified according to the strategy that their data were compatible with at date of PCI.
Treatment assignment	Before PCI, patients were randomly assigned to receive in an open‐label fashion either intravenous bivalirudin or intra‐arterial unfractionated heparin. Randomization was performed in a 1:1 ratio with stratification according to type of MI (STEMI or NSTEMI) and hospital.	Individuals randomized to a treatment strategy under PCI and were aware of the assigned strategy	Assignment was treated as if randomized within levels of the following baseline covariates: severity of MI (STEMI/NSTEMI, Killip class), center where PCI took place (university hospital/nonuniversity hospital), demographics (sex, age), lifestyle characteristics (weight), laboratory measurements (GFR), diagnoses (bleeding, anemia), and medications (warfarin, novel oral anticoagulants, P2Y12 inhibitors)
Outcomes	Composite of death from any cause, MI, or major bleeding events by 180 d Each separate component of the composite	Same as VALIDATE but outcomes identified as follows: Composite death from any cause identified from the Swedish Cause of Death register, MI identified from the SWEDEHEART register, and major bleeding events identified from the Swedish Inpatient and Outpatient registers or the SWEDEHEART register by 180 d	Same as target trial
Follow‐up	Starts at treatment assignment and ends at date of first outcome (separately for analysis of each outcome), migration, or 180 d after baseline	Same as VALIDATE apart from: Unable to identify migration date	Same as target trial
Causal contrast	Intention‐to‐treat effect	Intention‐to treat‐effect Per‐protocol effect	Observational analogue of the per‐protocol effect
Statistical analysis	Intention‐to‐treat analysis Kaplan–Meier plots Treatment differences were estimated with the use of the log‐rank test and Cox regression	Same intention‐to‐treat analysis as VALIDATE Per‐protocol analysis: Kaplan‐Meier plots adjusted for baseline covariates via inverse probability weighting Treatment differences were estimated with use of pooled logistic regression models with a flexible time‐varying intercept and product terms between the treatment and time. Baseline covariates adjusted for using 2 alternative approaches: inverse probability weighting (the baseline covariates were not included in the outcome model) and standardization (the baseline covariates were included in the outcome model).	Same as target trial

GFR indicates glomerular filtration rate; MI, myocardial infarction; NSTEMI, non–ST‐segment–elevation myocardial infarction; PCI, percutaneous coronary intervention; STEMI, ST‐segment–elevation myocardial infarction; SWEDEHEART, Swedish Web System for Enhancement and Development of Evidence‐Based Care in Heart Disease Evaluated According to Recommended Therapies; and VALIDATE, Bivalirudin Versus Heparin in ST‐Segment and Non–ST‐Segment Elevation Myocardial Infarction in Patients on Modern Antiplatelet Therapy.

### Trial Results

A total of 6006 patients underwent randomization in the VALIDATE trial, with 3004 assigned to bivalirudin and 3002 assigned to heparin. The risk of the composite end point did not differ between the treatment groups 30 days after PCI. By 180 days, the composite end point occurred in 12.3% of patients (369 of 3004) in the bivalirudin group and in 12.8% (383 of 3002) in the heparin group, with an HR of 0.96 (95% CI, 0.83 – 1.10). Death occurred in 2.9% of patients in the bivalirudin group and in 2.8% in the heparin group (without accounting for censoring), with an HR of 1.05 (95% CI, 0.78 – 1.41). MI occurred in 2.0% of patients in the bivalirudin group and in 2.4% in the heparin group, with an HR of 0.84 (95% CI, 0.60 – 1.19). Major bleeding occurred in 8.6% of patients in both the bivalirudin and heparin groups, with an HR of 1.00 (95% CI, 0.84 – 1.19).

## Specifying and Emulating a Target Trial in the SWEDEHEART Registry

### Data Sources

SWEDEHEART collects data from all patients hospitalized for acute coronary syndrome or undergoing coronary or valvular intervention for any indication in all relevant hospitals across Sweden.^13^ The registry was officially launched in 2009 when 4 existing cardiovascular healthcare quality registries were merged: RIKS‐HIA (Register of Information and Knowledge About Swedish Heart Intensive Care Admissions), SCAAR (Swedish Coronary Angiography and Angioplasty Registry), SEPHIA (Swedish Heart Surgery Register and the National Registry of Secondary Prevention), and the Swedish Heart Surgery Registry. SWEDEHEART was used to collect information for patients when they were randomized in the VALIDATE trial; hence, the target trial was emulated in the same population as the original trial and the data collection process was broadly similar between the 2 studies. SWEDEHEART is also linked to the Inpatient and Outpatient Register, which records all primary and secondary diagnoses and procedures from inpatient hospitalizations and outpatient specialist care visits across Sweden; the Swedish Cause of Death register, which records all deaths and causes of death; and the Prescribed Drug register, which collects information on all dispensed medications.^15^, ^16^, ^17^

The approach for emulating a target trial similar to the VALIDATE trial had 2 steps: (1) specifying the protocol of the target trial, and (2) emulating the target trial using the observational data from the SWEDEHEART registry. The first step is straightforward because our target trial has the same protocol as the VALIDATE trial, with exceptions only when the observational data were not adequate to identify the information collected in the trial. Table 1 summarizes the target trial protocol and outlines the emulation procedure described below.

### Eligibility Criteria

We identified individuals in the SWEDEHEART registry who met the target trial’s eligibility criteria. The eligibility criteria for the target trial were the same as the VALIDATE trial with 6 exceptions. First, the study period was between January 2012 to May 2014, which immediately precedes the period of the VALIDATE trial, as all eligible patients during that period were considered in the VALIDATE trial. Second, no informed consent was requested and therefore the target trial could not exclude individuals who would have not been asked or declined participation if asked. Third, the target trial used a proxy measure to indicate known terminal disease with life expectancy <1 year (palliative care; dialysis; dementia; or malignant disease, including pancreatic, lung, liver, stomach, and brain cancer, or mesothelioma). Fourth, some eligibility criteria could not be applied in the target trial because of unavailable data (ongoing bleeding, contraindications to study medication). Fifth, there was no information on the dosage of heparin before PCI, thus we excluded all individuals with any prior heparin use. Sixth, individuals who died on the day of PCI were excluded and identification of outcomes started from MI and bleeding occurred before or after PCI when the events occurred on the same day as the procedure.

### Treatment Strategies

The treatment strategies in the target trial closely mimicked those in the VALIDATE trial and were: (1) administration of bivalirudin, or (2) administration of heparin during PCI. The SWEDEHEART registry contains information on the anticoagulant given under PCI but does not contain information on dosage. In the target trial, we therefore assumed that if a patient was given both treatments, then the heparin was low‐dose, and the treatment strategy was defined as bivalirudin.

### Treatment Assignment

We classified eligible individuals in the SWEDEHEART registry into 2 groups according to the strategy their data were compatible with at baseline. That is, our emulation presumed that all individuals assigned to a treatment strategy ended up receiving it. Our emulation treated individuals as if they were randomly assigned to a treatment strategy conditional on the baseline covariates: severity of MI (STEMI/NSTEMI, Killip class, angiography finding), center where the PCI took place (university hospital/nonuniversity hospital), demographics (sex, age), lifestyle characteristics (weight, smoking), laboratory measurements (glomerular filtration rate, heart rate, systolic blood pressure, diastolic blood pressure), diagnoses (diabetes mellitus, severe bleeding, anemia), medications (warfarin, antihypertensives, lipid‐lowering treatment), and prior cardiovascular disease or cardiovascular procedures (MI, PCI, coronary artery bypass grafting). See Table S1 for further details on covariates and their definitions.

### Outcomes

The outcomes in the target trial were the same as the VALIDATE trial and included death from any cause, MI, major bleeding events, and a composite of all of the above outcomes. Death was identified from the Cause of Death register, MI from the SWEDEHEART registry, and bleeding from the Inpatient and Outpatient register or SWEDEHEART registry (full outcome definitions in are shown in Table S2).

### Follow‐Up

Each individual was followed from the day after PCI until the outcome of interest or 180 days, whichever occurred first. In the MI and bleeding analyses, individuals were not censored if they died during follow‐up, which is valid when estimating the total effect of treatment, but we performed sensitivity analysis to explore censoring at death (Sensitivity 10).^18^ Outcome data on individuals who migrated out of Sweden were unavailable, but the probability of migration during such a short period is low.

### Causal Contrast

In the target trial, the intention‐to‐treat effect was the effect of being assigned to bivalirudin versus heparin at baseline and the per‐protocol effect was the effect of receiving the assigned treatment. Our emulation focused on an observational analog of the per‐protocol effect. In the VALIDATE trial there was 0.5% crossover between treatment groups, so the intention‐to‐treat effect was approximately equal to the per‐protocol effect.

### Statistical Analysis

We estimated the 14‐, 30‐, and 180‐day risk (cumulative incidence), risk difference (RD), and risk ratio (RR) for each outcome using pooled logistic regression models with a flexible time‐varying intercept and product terms between the treatment and time.^19^ We adjusted for baseline covariates using 2 alternative approaches: inverse probability weighting and outcome regression followed by standardization. We truncated the stabilized inverse probability weights at their 99th percentile to prevent outliers with extreme weights from influencing effect estimates. We plotted unweighted and inverse probability–weighted Kaplan‐Meier survival curves for all outcomes. We used nonparametric bootstrapping with 500 samples to calculate all 95% CIs. Further details on our modelling approach are in Data S1.

We repeated the 180‐day analysis using inverse probability–weighted models stratified by MI type (STEMI/NSTEMI) to explore effect modification. Finally, we performed several sensitivity analyses using inverse probability–weighted models to assess the sensitivity of the eligibility criteria, treatment strategies, outcomes, confounders, follow‐up, and analysis. Full details on sensitivity analyses are in Data S2.

### Data, Materials, and Code Disclosure

All analysis codes are available at: https://github.com/tonymatthews/validate. Pseudonymized personal data were obtained from national Swedish Registry holders after ethical approval and secrecy assessment. According to Swedish laws and regulations, personal sensitive data can only be made available for researchers who fulfill legal requirements for access to personal sensitive data.

## Results

**Figure 1:**
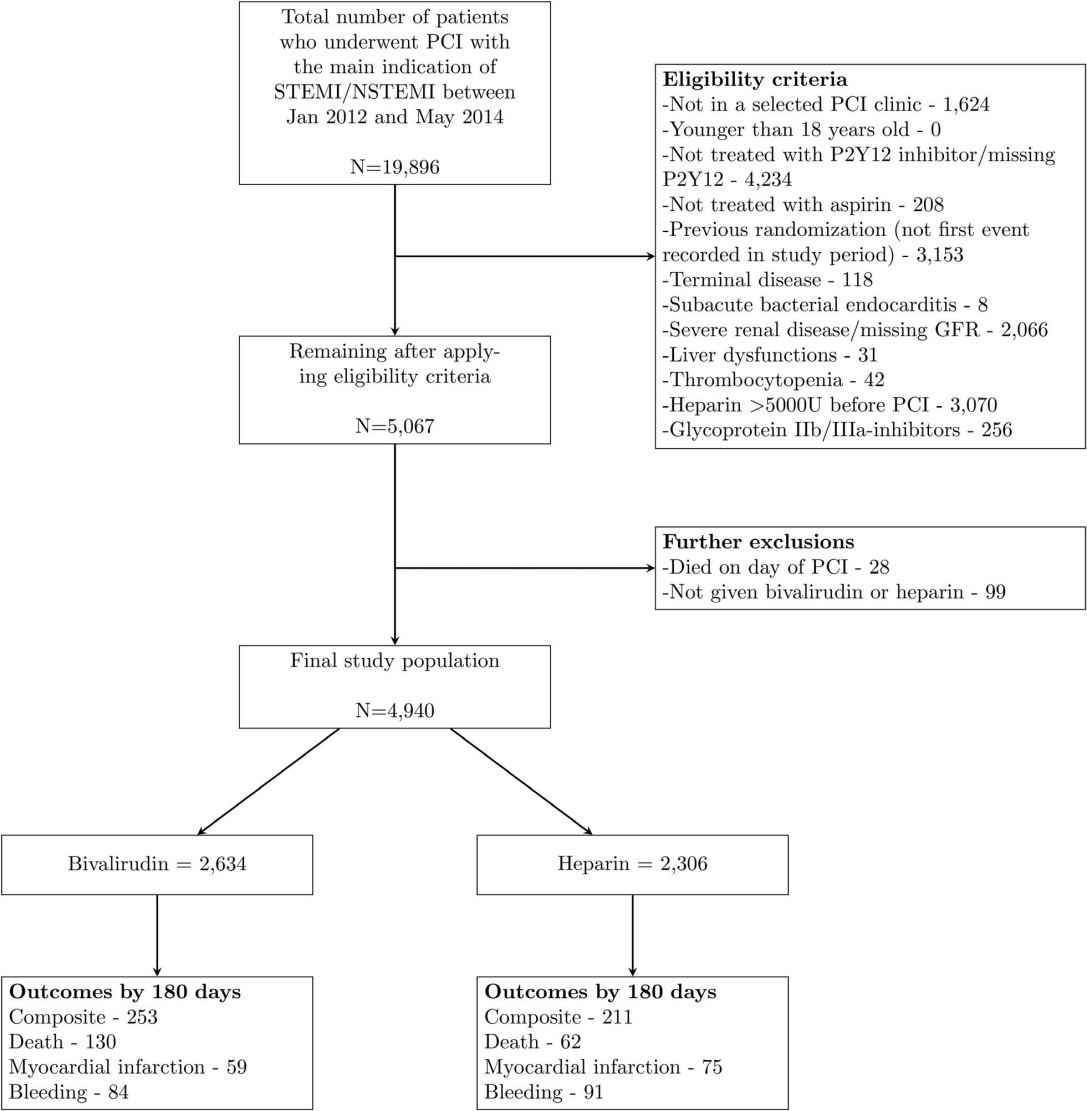
Flowchart of individuals eligible for the observational emulation of a target trial of bivalirudin vs heparin, SWEDEHEART (Swedish Web System for Enhancement and Development of Evidence‐Based Care in Heart Disease Evaluated According to Recommended Therapies) register, 2012 to 2014. NSTEMI indicates non–ST‐segment–elevation myocardial infarction; PCI, percutaneous coronary intervention; and STEMI, ST‐segment–elevation myocardial infarction.

Figure 1 shows a flowchart of patient selection for the target trial emulation. Table 2 shows the baseline characteristics of the 4940 eligible patients, of whom 2634 were given bivalirudin and 2306 heparin. Compared with those in the heparin group, patients in the bivalirudin group were more likely to be diagnosed with STEMI over NSTEMI before PCI (81% versus 17%).

**Table 2:** Baseline Characteristics of Eligible Individuals for the Observational Emulation of a Target Trial of Bivalirudin Versus Heparin, SWEDEHEART Register, 2012 to 2014

	Bivalirudin	Heparin
No.	2634	2306
Women, n (%)	731 (27.8)	652 (28.3)
Age, median (IQR), y	68 (59–76)	69 (61–76)
University hospital, n (%)	1074 (40.8)	995 (43.1)
STEMI, n (%)	2131 (80.9)	398 (17.3)
Killip class, n (%)		
1	2413 (91.6)	1894 (82.1)
2	96 (3.6)	34 (1.5)
3	21 (0.8)	8 (0.3)
4	26 (1.0)	5 (0.2)
Missing	78 (3.0)	365 (15.8)
Angiography finding, n (%)		
Normal	3 (0.1)	50 (2.2)
1 Vessel + no left main	1365 (51.8)	1135 (49.2)
2 Vessels + no left main	720 (27.3)	701 (30.4)
3 Vessels + no left main	426 (16.2)	330 (14.3)
Left main	120 (4.6)	90 (3.9)
Heart rate, median (IQR), beats per min	75 (63–89)	76 (66–89)
Missing, n (%)	98 (3.7)	67 (2.9)
Systolic blood pressure, median (IQR), mm Hg	144 (125–165)	155 (140–174)
Missing, n (%)	99 (3.8)	67 (2.9)
Diastolic blood pressure, median (IQR), mm Hg	85 (75–97)	88 (79–99)
Missing, n (%)	279 (10.6)	144 (6.2)
Anemia severity category, n (%)		
Severe	2 (0.1)	3 (0.1)
Moderate	91 (3.5)	42 (1.8)
Mild	318 (12.1)	211 (9.2)
No anemia	2039 (77.4)	1836 (79.6)
Missing	184 (7.0)	214 (9.3)
Hemoglobin, median (IQR), g/L	142 (131–152)	144 (133–153)
Weight, median (IQR), kg	80 (70–90)	81 (72–92)
Smoking status, n (%)		
Never	959 (36.4)	895 (38.8)
Ex‐smoker (>1 mo)	776 (29.5)	843 (36.6)
Current	736 (27.9)	522 (22.6)
Missing	163 (6.2)	46 (2.0)
Prior MI, n (%)	406 (15.4)	519 (22.5)
Prior PCI, n (%)	323 (12.3)	426 (18.5)
Prior coronary artery bypass grafting, n (%)	115 (4.4)	185 (8.0)
Prior serious bleeding, n (%)	87 (3.3)	112 (4.9)
Diabetes mellitus, n (%)	429 (16.3)	459 (19.9)
Kidney function (GFR), median (IQR)	87 (65–112)	85 (64–107)
Hypertension treatment, n (%)	1256 (47.7)	1343 (58.2)
Lipid‐lowering treatment, n (%)	733 (27.8)	1035 (44.9)
Warfarin, n (%)	27 (1.0)	40 (1.7)
Outcomes		
Composite outcome (30 d), n (%)	140 (5.3)	83 (3.6)
Composite outcome (180 d), n (%)	253 (9.6)	211 (9.2)
Death (30 d), n (%)	86 (3.3)	22 (1.0)
Death (180 d), n (%)	130 (4.9)	62 (2.7)
MI (30 d), n (%)	27 (1.0)	27 (1.2)
MI (180 d), n (%)	59 (2.2)	75 (3.3)
Bleeding (30 d), n (%)	32 (1.2)	37 (1.6)
Bleeding (180 d), n (%)	84 (3.2)	91 (3.9)

GFR indicates glomerular filtration rate; IQR, interquartile range; MI, myocardial infarction; PCI, percutaneous coronary intervention; STEMI, ST‐segment–elevation myocardial infarction; and SWEDEHEART, Swedish Web System for Enhancement and Development of Evidence‐Based Care in Heart Disease Evaluated According to Recommended Therapies.

Table 3 shows the estimated 180‐day risks, RD, and RRs obtained via inverse probability weighting and standardization. The inverse probability–weighted risk of the composite outcome was 9.3% (95% CI, 8.2% – 10.4%) in the bivalirudin group and 10.0% (95% CI, 8.7% – 11.3%) in the heparin group, which results in a RD of −0.7% (95% CI, −2.5% to 1.1%) and an RR of 0.93 (95% CI, 0.77 – 1.12). The risk of death was 4.1% (95% CI, 3.4% – 4.8%) in the bivalirudin group and 3.4% (95% CI, 2.5% – 4.2%) in the heparin group, which results in a risk difference of 0.7% (95% CI, −0.4% to 1.8%) and RR of 1.21 (95% CI, 0.88 – 1.68). The risk of MI was 3.0% (95% CI, 2.3% – 3.8%) in the bivalirudin group and 2.8% (95% CI, 2.2% – 3.4%) in the heparin group, which results in a RD of 0.2% (95% CI, −0.8% to 1.2%) and RR of 1.08 (95% CI, 0.76 – 1.54). The risk of bleeding was 3.2% (95% CI, 2.5% – 3.9%) in the bivalirudin group and 4.6% (95% CI, 3.7% – 5.6%) in the heparin group, which results in a RD of −1.4% (95% CI, −2.6% to −0.3%) and an RR of 0.69 (95% CI, 0.50 – 0.95). Standardized estimates were similar.

**Table 3:** Estimated 180‐d Risk, Risk Difference, and RRs from the Observational Emulation* of a Target Trial of Bivalirudin Versus Heparin, SWEDEHEART Register, 2012–2014

Outcome	Risk, % (95% CI)		Risk Difference, % (95% CI)	Risk Ratio (95% CI)
	Bivalirudin	Heparin		
Inverse‐probability weighted				
Composite	9.3 (8.2 to 10.4)	10.0 (8.7 to 11.3)	−0.7 (−2.5 to 1.1)	0.93 (0.77 to 1.12)
Death	4.1 (3.4 to 4.8)	3.4 (2.5 to 4.2)	0.7 (−0.4 to 1.8)	1.21 (0.88 to 1.68)
MI	3.0 (2.3 to 3.8)	2.8 (2.2 to 3.4)	0.2 (−0.8 to 1.2)	1.08 (0.76 to 1.54)
Bleeding	3.2 (2.5 to 3.9)	4.6 (3.7 to 5.6)	−1.4 (−2.6 to −0.3)	0.69 (0.50 to 0.95)
Standardized				
Composite	9.2 (7.5 to 10.8)	9.9 (8.0 to 11.9)	−0.8 (−3.6 to 2.0)	0.92 (0.74 to 1.14)
Death	4.3 (3.2 to 5.4)	3.4 (2.1 to 4.6)	1.0 (−0.8 to 2.7)	1.28 (0.92 to 1.80)
MI	2.8 (1.6 to 3.9)	2.7 (1.7 to 3.7)	0.1 (−1.7 to 1.8)	1.03 (0.66 to 1.59)
Bleeding	3.0 (1.9 to 4.1)	4.3 (2.6 to 5.9)	−1.3 (−3.6 to 1.1)	0.70 (0.46 to 1.09)

MI indicates myocardial infarction; and SWEDEHEART, Swedish Web System for Enhancement and Development of Evidence‐Based Care in Heart Disease Evaluated According to Recommended Therapies.

* Adjusted at baseline for severity of MI (STEMI/NSTEMI, Killip class, angiography finding), center where percutaneous coronary intervention (PCI) took place (university hospital/nonuniversity hospital), demographics (sex, age), lifestyle characteristics (weight, smoking), laboratory measurements (glomerular filtration rate, heart rate, systolic blood pressure, diastolic blood pressure), diagnoses (diabetes mellitus, severe bleeding, anemia), medications (warfarin, antihypertensives, lipid lowering treatment), and prior cardiovascular disease or cardiovascular procedures (MI, PCI, coronary artery bypass grafting).

**Figure 2:**
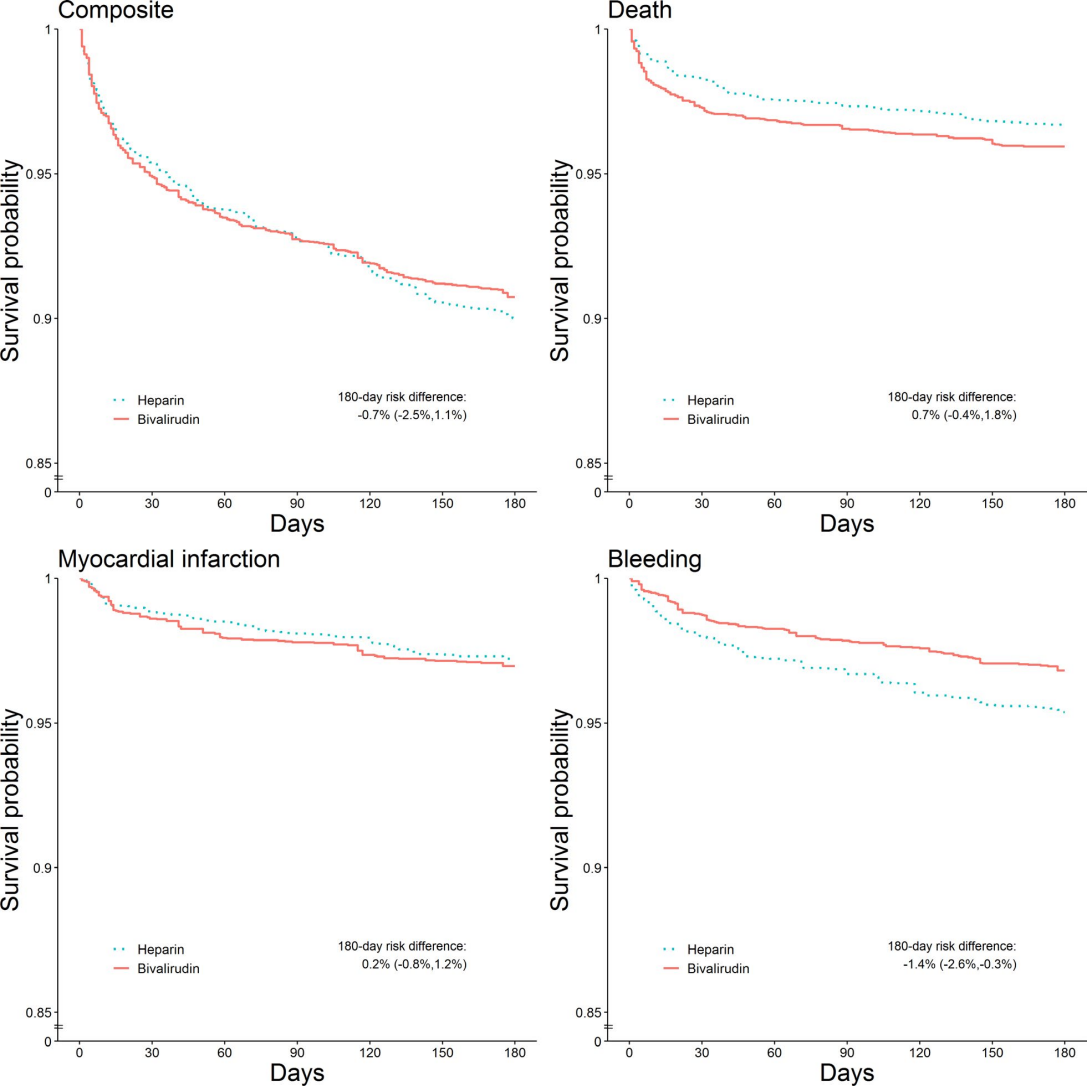
Inverse‐probability weighted survival curves from an observational emulation of a target trial of bivalirudin vs heparin, SWEDEHEART (Swedish Web System for Enhancement and Development of Evidence‐Based Care in Heart Disease Evaluated According to Recommended Therapies) register, 2012–2014. Adjusted at baseline for severity of myocardial infarction (MI; STEMI/NSTEMI, Killip class, angiography finding), center where percutaneous coronary intervention (PCI) took place (university hospital/nonuniversity hospital), demographics (sex, age), lifestyle characteristics (weight, smoking), laboratory measurements (glomerular filtration rate, heart rate, systolic blood pressure, diastolic blood pressure), diagnoses (diabetes mellitus, severe bleeding, anemia), medications (warfarin, antihypertensives, lipid‐lowering treatment, and prior cardiovascular disease or cardiovascular procedures (MI, PCI, coronary artery bypass grafting).

Figure 2 shows the inverse probability–weighted survival curves (unweighted survival curves in Figure S2). The MI‐free survival almost overlapped throughout the 180 days of follow‐up. However, there was an elevated risk of death in the bivalirudin group compared with the heparin group in the 14 days after PCI. The short‐term difference in risk of death can also be seen in the estimated risk difference and RR by 14 and 30 days (Tables S3 and S4).

Table S5 shows the 180‐day risks, RDs, and RRs obtained via inverse probability weighting, stratified by MI type, and there were no apparent differences between strata. Tables S6 to S15 and Figure S2 show results from sensitivity analyses; all results were broadly similar to those estimated in the main analyses.

## Discussion

We emulated a target trial similar to the VALIDATE trial using real‐world observational data from SWEDEHEART, the same register in which the trial was undertaken. There was broad agreement in the estimates for death and MI by 180 days from the trial and the observational emulation of the target trial; both found little differences in risk between patients given heparin and bivalirudin. However, there were some important differences between the estimates from the VALIDATE trial and its observational emulation.

First, the target trial could not define a bleeding outcome similar to that in the VALIDATE trial, which was ascertained using a combination of phone calls to patients at 7 and 180 days after PCI and a review of hospital records from registers. Exclusively using the registers, as was done in the target trial, only allowed us to identify the most severe cases of bleeding. We also attempted to expand the bleeding outcome in a sensitivity analysis (outcome definition in Table S2 and results in Table S12), but we were still unable to fully capture less severe cases of bleeding. This difference in the definition of bleeding is likely responsible for the lower risk estimates for the bleeding and the composite outcomes in the VALIDATE trial and target trial emulation.

Second, the observational estimates showed a RD of 1.0% (95% CI, 0.1% ​– 1.8%) for death after 14 days of follow‐up (Figure 2, Table S3), whereas the trial estimates showed no discernible difference in mortality throughout the entire follow‐up. This almost instantaneous difference in mortality may be attributable to prognostic factors that were imbalanced between treatment groups, and unadjusted for, but that had less impact over the entire follow‐up period. It is possible, for example, that patients perceived to be at high risk of bleeding, and hence at higher risk of early death, may have been more likely to be administered bivalirudin (because bivalirudin was marketed as having a lower risk of bleeding than heparin). Another explanation is that the data available on severity of MI were not granular enough to fully account for the fact that individuals administered bivalirudin were more likely to be severely ill or have a poor early prognosis, as reflected, for example, by the higher proportion of patients diagnosed with STEMI in the bivalirudin compared with the heparin group. Even with rich data from the SWEDEHEART register, we were not able to accurately identify these potentially important characteristics. One way to aid future researchers in identifying significant potential confounders would be if registers included more detailed information on reasons for making treatment decisions. Another possible, but perhaps less likely, explanation for this difference in the short‐term risk of death is the inability to emulate a target trial that would have precisely the same eligibility criteria as the index trial; we discuss this issue in more detail below.

The number of individuals randomized in the VALIDATE trial was 6006 (2971 individuals either declined, were not asked, or could not be asked to participate), whereas the number of individuals included in the target trial emulation over a recruitment period of similar length was 4940. This discrepancy is partly because of our inability to emulate the eligibility criteria for the VALIDATE trial. Individuals given >5000 U heparin before PCI in the VALIDATE trial were excluded, but the SWEDEHEART data do not include details on the dosage of heparin administered. We, therefore, excluded all 3070 individuals given any dose of heparin before PCI, of whom some would have received low doses. A sensitivity analysis that did not exclude any patient given heparin before PCI (Table S9) included 7652 individuals, and results for death and MI by 180 days were broadly similar to the results from primary analyses. The RD for death between patients given bivalirudin and heparin early in follow‐up was, however, reduced in this sensitivity analysis, as shown in the inverse probability–weighted Kaplan‐Meier survival curves (Figure S2). This suggests that by excluding patients given any prior heparin in the main analysis, rather than only those given high‐dose heparin as in the trial, we may be selecting a group of individuals given heparin during PCI who are at lower risk of death soon after start of follow‐up compared with those in the trial. Results from this sensitivity analysis demonstrate the importance of designing a target trail that closely mimics the eligibility criteria of the index trial if aiming for comparable results, and, as mentioned, is another possible explanation for the differences in short‐term risk of death discussed above.

The SWEDEHEART register contains some of the most complete and rich routinely collected data available worldwide when a patient undergoes PCI. Even with high‐quality data, however, close harmonization of protocols, adjustment for important confounders, and analytic methods appropriate for estimating causal quantities analogous to those estimated in the trial, we have shown that it is not always possible for the target trial emulation to obtain the same results as an index trial attributable to trials collecting more detailed, study‐specific information that are not routinely collected. Nevertheless, we have shown that using real‐world data to emulate a target trial can deliver accurate effect estimates for this specific clinical question. It is, therefore, possible to foresee a system in which data from trials with short‐term follow‐up are combined with real‐world data to estimate the effects of certain interventions over longer periods of follow‐up.^20^ Estimating the effect from the short trial would overcome the potential problem of confounding soon after the intervention. We could then use observational data to estimate the effect during the remainder of follow‐up and create synthetic survival curves using both data sources. This would enable prompt estimation of effects of interventions over longer periods of follow‐up and allow adaptive clinical or regulatory changes to be put quickly into practice. Synergy between trials and real‐world data has the potential to revolutionize both the length of time patients can be followed up and the speed at which results from trials reach clinical practice.

## Sources of Funding

This work was supported by a grant from the Swedish Research Council (registration number 2018‐03028).

## Disclosures

Dr Matthews reports grants from FORTE during the conduct of the study; Dr Dahabreh reports grants from PCORI (award ME‐1502‐27794) during the conduct of the study; Dr Erlinge reports personal fees from AstraZeneca, outside the submitted work; Dr Feychting, Dr Berglund, and Dr Hernan report grants from the Swedish Research Council during the conduct of the study; and Dr Hernán reports personal fees from Cytel and ProPublica during the conduct of the study. The remaining authors have no disclosures to report.

## Supporting information

Data S1–S2
Tables S1–S15
Figures S1–S2
